# The Association of Postoperative Complications and Hospital Costs Following Distal Pancreatectomy

**DOI:** 10.3389/fsurg.2022.890518

**Published:** 2022-05-30

**Authors:** Laurence Weinberg, Vidhura Ratnasekara, Anthony T. Tran, Peter Kaldas, Tom Neal-Williams, Michael R. D’Silva, Jackson Hua, Sean Yip, Patryck Lloyd-Donald, Luke Fletcher, Ronald Ma, Marcos V. Perini, Mehrdad Nikfarjam, Dong-Kyu Lee

**Affiliations:** ^1^ Department of Anaesthesia, Austin Health, Heidelberg, Australia; ^2^ Department of Critical Care, The University of Melbourne, Austin Health, Heidelberg, Australia; ^3^ Department of Surgery, The University of Melbourne, Austin Health, Heidelberg, Australia; ^4^ Data Analytics Research and Evaluation (DARE) Centre, Austin Health, Heidelberg, Australia; ^5^ Business Intelligence Unit, Austin Health, Heidelberg, Australia; ^6^ Department of Anesthesiology and Pain Medicine, Dongguk University Ilsan Hospital, Goyang, Republic of Korea

**Keywords:** distal pancreatectomy (DP), distal pancreatectomy and splenectomy, complications, costs, anaesthesia, surgery

## Abstract

**Background:**

Understanding the financial implications associated with the complications post-distal pancreatectomy (DP) may be beneficial for the future optimisation of postoperative care pathways and improved cost-efficiency. The primary outcome of this retrospective study was the characterisation of the additional cost associated with postoperative complications following DP. The secondary outcome was the estimation of the prevalence, type and severity of complications post-DP and the determination of which complications were associated with higher costs.

**Methods:**

Postoperative complications were retrospectively examined for 62 adult patients undergoing distal pancreatectomy at an Australian university hospital between January 2012 and July 2021. Complications were defined and graded using the Clavien–Dindo (CVD) classification system. In-hospital cost of index admission was calculated using an activity-based costing methodology and was reported in US dollars at 2021 rates. Regression modelling was used to investigate the relationships among selected perioperative variables, complications and costs.

**Results:**

45 patients (72.6%) experienced one or more postoperative complications. The median (IQR) hospital cost in US dollars was 31.6% greater in patients who experienced complications compared to those who experienced no complications ($40,717.8 [27,358.0–59,834.3] vs. $30,946.9 [23,910.8–46,828.1]). Costs for patients with four or more complications were 43.5% higher than for those with three or fewer complications (*p* = 0.015). Compared to patients with no complications, the median hospital costs increased by 17.1% in patients with minor complications (CVD grade I/II) and by 252% in patients who developed major complication (i.e., CVD grade III/IV) complications.

**Conclusion:**

Postoperative complications are a key target for cost-containment strategies. Our findings demonstrate a high prevalence of postoperative complications following distal pancreatectomy with number and severity of postoperative complications being associated with increased hospital costs. (Registered in the Australian New Zealand Clinical Trials Registry [No. ACTRN12622000202763]).

## Introduction

The incidence of pancreatic cancer is increasing (1–4). Given that surgically resectable lesions of the pancreas improve five-year post-diagnosis survival rates (5), the demand for and utility of distal pancreatectomy (DP) is also expected to rise proportionally. Despite the benefits of a distal pancreatic resection, there are various complications that have been documented during the postoperative period (1, 6, 7). The sequelae of a post-surgical complication can lead to clinical pathology that may require readmission to hospital (1) and confer poorer long-term prognosis for a patient (8). Additionally, there is evidence suggesting that DP has a higher readmission rate than other pancreatic resection techniques (9).

Surgical complications following DP can result in added costs and increased use of resources for hospitals. Understanding the financial implications associated with the complications post-DP may be beneficial for the future optimisation of postoperative care pathways and improved cost-efficiency. Although several studies have investigated the financial consequences of complications for various surgeries (10–15), few have detailed the relationship between complication incidence, severity, and cost ramifications specific to DP.

We conducted a retrospective study to analyse the complications associated with DP and the related costs. Our primary aim was to characterise the relationship between postoperative complications and costs incurred by the health organisation. The secondary aim was to estimate the prevalence of the type and severity of complications and stratify which complications contribute the most to escalating costs. We hypothesised that hospital costs would be positively correlated with an increasing number and severity of postoperative complications following DP.

## Materials and Methods

### Study Design

A single-centre retrospective cohort study was conducted to better characterise the postoperative complications following DP and the associated costs. The Human Research Ethics Committee at Austin Health (HREC 19/Austin/88) approved this study and provided a waiver for participant consent. The study protocol was retrospectively registered in the Australian New Zealand Clinical Trials Registry (No. ACTRN12622000202763, accessible from https://www.anzctr.org.au/ACTRN12622000202763.aspx). This study is reported in accordance with the Strengthening the Reporting of Cohort Studies in Surgery (STROCSS) guidelines (16).

### Setting and Cohort

This study was conducted at Austin Health, a large public university teaching hospital in Victoria, Australia with a high volume of hepatobiliary and pancreatic surgery. Patients were identified using the *International Statistical Classification of Diseases and Related Health Problems, 10th Revision* and codes specific to DP.

We included adult patients aged 18 years or above who had undergone elective open or laparoscopic DP between January 2012 and July 2021. We excluded patients who had undergone total or central pancreatectomy, pancreaticoduodenectomy, and DP secondary to another major procedure (e.g., pelvic exenteration, cystectomy or DP secondary to trauma). Laparoscopic and open procedures were included. Austin Health does not provide robotic surgical services for DP.

### Outcomes

The primary outcome was the characterisation of the additional cost associated with postoperative complications following DP. The secondary outcome was the estimation of the prevalence, type and severity of complications post-DP and the determination of which complications were associated with higher costs.

### Data Sources

Prospectively recorded data, including preoperative, intraoperative and postoperative details, were collected from the Austin Health Cerner electronic health records (Cerner Millenium, Kansas, USA). The perioperative data collected consisted of patient demographics, such as gender, height, weight and the age-adjusted Charlson Comorbidity Index (CCI).

The preoperative laboratory data collected included haemoglobin, platelet and white blood cell counts, serum electrolytes (i.e., sodium, potassium, chloride and bicarbonate), serum creatinine, serum urea level and the estimated glomerular filtration rate (eGFR). The collected intraoperative data included fluids transfused, medications administered and haemodynamic parameters. Postoperative data comprised equivalent laboratory data to that for the preoperative variables in addition to arterial blood gas (ABG) analysis, including pH, pCO_2_, pO_2_, base excess and lactate concentration.

Data was collected on postoperative high dependency unit (HDU) and intensive care unit (ICU) admissions, HDU and ICU care duration, length of hospital stay, medical emergency team (MET) calls, planned and unplanned 30-day readmission and days alive and out of hospital at 90 days postoperatively. All reported postoperative complications were collected and recorded as a number of complications and the highest severity based on the Clavien–Dindo (CVD) grade for each patient.

### Definitions

Total hospital cost was defined as the sum of direct and indirect in-hospital costs of index admission for DP. Raw costing data were obtained from Austin Health’s business intelligence unit and costing centre. This data included patient care activities relating to anaesthesia, the operative theatre, ICU admission, ward, medical consultations, allied health, pathology, blood products, pharmacy, radiology, MET calls and hospital in the home. Costs incurred during the preoperative period were excluded from the data analysis to prevent potential confounding factors due to preoperative cost drivers. In-hospital costs arising from unplanned readmissions within 30 days of discharge were included in the total cost. Costs were inflated to 31 August 2021 levels based on the end-of-fiscal-quarter Australian consumer price index and converted to US dollars based on the market rate on 31 August 2021. In-hospital costs were calculated according to an activity-based costing methodology that allocated costs based on service volume.

Postoperative complications were defined as any deviation from the normal expected postoperative course, as guided by the European Perioperative Clinical Outcome definitions (17). The severity of complications was graded according to the Clavien-Dindo (CVD) classification system (18). This validated system categorises complication severity based on the level of treatment required. CVD grade I complications include any deviation from the normal postoperative course that does not require intervention, excluding analgesia, antipyretics, antiemetics, diuretics, electrolyte replacement and physiotherapy. CVD grade II complications include those that require pharmacological intervention, blood transfusion or total parenteral nutrition. CVD grade III complications require radiological, endoscopic, or surgical intervention. CVD grade IV complications include life-threatening complications that require intensive care management, and CVD grade V represents patient death. Patients were stratified into groups based on the most severe complication recorded. Postoperative complications during index admission were extracted from electronic medical records by the Data Analytics Research and Evaluation (DARE) Centre and were independently cross-checked with a complete chart review by two authors.

The length of hospital stay was defined as the number of days from completion of surgery to discharge. Readmissions were specified as any planned or unplanned readmission within 30 days post-discharge. Mortality was defined as inpatient mortality, as outlined in the CVD system.

### Statistical Analysis Methods

The statistical analysis was performed using IBM SPSS Statistics for Windows, version 23 (IBM Corp., 2015, Armonk, NY, USA) and R, version 4.1.2 (R Development Core Team, Vienna, Austria, 2021). All continuous variables were tested for normality using a quantile-quantile plot. When a variable violated the normality assumption, non-parametric statistical methods were considered. Missing data analysis was performed to detect variables with a missing rate greater than 5%. Any clinically and statistically meaningful variables with a missing rate higher than 5% were subjected to multiple imputations to the corresponding variable if the missing patterns were completely at random. For variables with a missing rate of less than 5%, cases were excluded during analysis.

Unadjusted hospital cost analysis was performed for postoperative complications. According to the presence, number and severity of complications, and surgical techniques, hospital costs were compared using the Wilcoxon rank-sum test and the Kruskal–Wallis one-way analysis of variance on ranks. Dunn’s all pairwise multiple comparison procedures were used as a post-hoc test when the null hypothesis of the Kruskal–Wallis test was rejected.

Adjusted hospital cost analysis was performed in two steps. First, Pearson’s or Spearman’s correlation analysis was used to determine the variables relevant to hospital costs. Variables were selected for further evaluation if the correlation coefficient was statistically significant, and the absolute value was greater than 0.2. Among the selected variables, the adjusting factors were chosen according to the clinical significance and correlation between the selected variables. Second, linear regression modelling was applied to determine the effects of postoperative complications on hospital costs. The autocorrelation of hospital costs was evaluated using Durbin–Watson statistics. Multicollinearity between covariates was tested with the variance inflation factor and collinearity diagnostics using the eigenvalues. Homoscedasticity was assessed using residual plots. Cook’s distance was also evaluated to detect outliers.

Data are presented as the mean ± standard deviation or as the median (interquartile range (IQR), [minimum : maximum]) for continuous variables and as a number (percentage) for categorical variables. Statistically inferred results are presented with 95% confidence intervals (CI) and *p-*values. A two-tailed *p-*value below 0.050 was considered statistically significant, with *p*-values adjusted using Bonferroni’s correction method as required.

## Results

### Baseline Patient Characteristics

**Figure 1:**
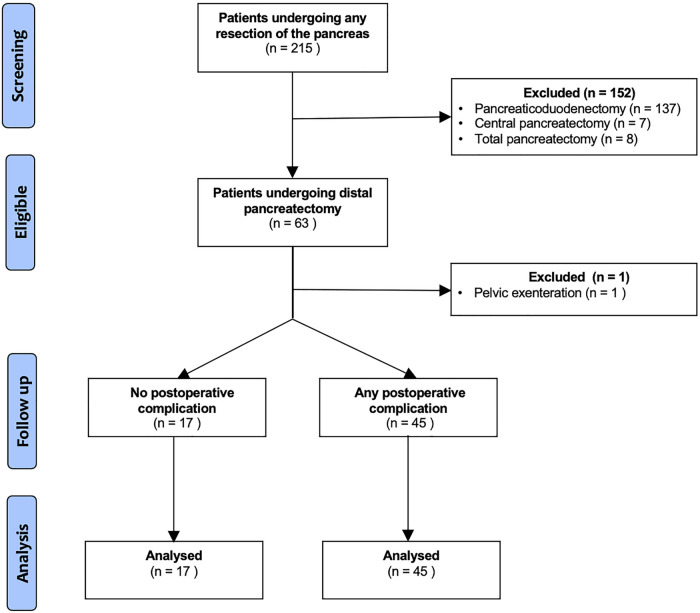
Flow diagram.

Of 215 potentially eligible patients who had undergone pancreatic resection at our institution, 152 were excluded. The reasons for exclusion included pancreaticoduodenectomy (*n* = 137), central pancreatectomy (*n* = 7), total pancreatectomy (*n* = 8) and DP secondary to another major surgical procedure (*n* = 1). A flow diagram is presented in Figure 1. In total, 62 patients were included in the final statistical analysis. Fourteen patients (22.6%) underwent laparoscopy surgery, of which six patients had a spleen preserving procedure. Forty-eight patients (77.4%) underwent open surgery, of which fifteen patients had a spleen preserving procedure. There was no conversion from laparoscopic to open surgery.

Among the data for the 62 patients, missing data analysis accounted for less than 5% of the missing values for all variables, except in the case of intraoperative diastolic blood pressure (24.2%). The variables with the next highest missing data rates were “postoperative ABG analysis” (3.2%) and “preoperative electrolytes” (1.6%). Statistical analysis was performed as a complete case analysis. The mean patient age was 58.2 ± 14.4 years (range: 24 to 83 years), and 39 patients (62.9%) were women. The mean weight and height of patients were 74.9 ± 18.6 kg and 167.0 ± 8.8 cm, respectively. The mean age-adjusted CCI was 4.7 ± 3.0. Additional preoperative patient data are presented in Table 1.

**Table 1:** Baseline patient characteristics, perioperative laboratory findings and postoperative outcomes.

Preoperative		Intraoperative		Postoperative	
Variables	Values	Variables	Values	Variables	Values
***Patient characteristics***		***Fluid use and transfusion***		***Laboratory values on arrival to RHDU***	
Female	39 (62.9%)	Blood loss (mL)	500 (275–1,550) [200:1,700]	Haemoglobin (g/L)	97.9 ± 16.1
Age (year)	58.2 ± 14.4	Urine output (mL)	248.7 ± 401.7	White cell (×10^9^/L)	18.4 ± 5.5
Weight (kg)	74.9 ± 18.6	Blood transfusion (number of patients receiving)	3 (4.8%)	eGFR (mL/min/1.73 m^2^)	75.2 ± 18.2
Height (cm)	167.0 ± 8.8	Red blood cell volume if patient received a blood transfusion (mL)	2,000 (273–543) [273:4,500]	Creatinine (mmol/L)	76 (67–90.25) [45:200]
Age adjusted CCI	4.7 ± 3.0	Crystalloid therapy (number of patients receiving)	61 (100%)	***Arterial blood gas analysis on arrival to post-anaesthesia recovery unit***	
***Laboratory findings***		Crystalloid volume administered (mL)	2,000 (1,000–3,000) [200:6,000]	pH, minimum	7.312 ± 0.063
Haemoglobin (g/L)	134.3 ± 14.5	Albumin (number of patients receiving)	20 (32.3%)	pCO_2_ (mmHg), maximum	49.1 ± 9.4
White cell (×10^9^/L)	7.10 (5.78–8.60) [2.90:19.60]	Albumin volume administered (mL)	250 (100–500) [100:1,000]	Base excess (mmol/L), minimum	−3.4 ± 3.3
HbA_1c_ (%)	5.6 (5.3–6.2) [4.8:11.8]	***Use of vasoactive medications***		Lactate (mmol/L), maximum	2.2 ± 1.3
Creatinine (mmol/L)	72.8 ± 18.9	Metaraminol (number of patients receiving)	41.0 (66.1)	***Postoperative destination***	
eGFR (mL/min/1.73 m^2^)	89.0 (77.8–90.0) [42.0:91.0]	Metaraminol (mg) (dose administered)	3.0 (1.5–8.25) [0.5:19.0]	HDU admission	34 (54.8%)
Ferritin (µ/L)	88 (35–183) [6:2,254]	Noradrenaline (number of patients receiving)	11 (17.7)	HDU admission time (hour)	7.4 ± 7.6
INR	1.0 ± 0.2	Noradrenaline (mg) (dose administered)	1.8 ± 2.5	ICU admission	11 (17.7%)
		Ephedrine (number of patients receiving)	17 (27.4)	ICU admission time (hour)	20.9 (17.6–106.2) [8.4:381.4]
		Ephedrine (mg) (dose administered)	13.6 ± 10.0	***Readmissions***	
		Phenylephrine (number of patients receiving)	2 (3.2)	Total number of events	Total number of events
		Phenylephrine (mg) (dose administered)	2.2 (1.9–) [1.9:2.5]	Related to surgery, planned	Related to surgery, planned
		Adrenaline (number of patients receiving)	1 (1.6)	Related to surgery, unexpected	Related to surgery, unexpected
		Adrenaline (ug) (dose administered)	15 (15–) [15:15]	Not related to surgery	Not related to surgery
		Clonidine (number of patients receiving)	8 (12.9)		
		Clonidine (ug) (dose administered)	67.5 (48.8–75.0) [7.5:100.0]		
		***Haemodynamic variables***			
		Hypotension^a^ (number of patients)	54 (87.1%)	* *	* *
		Number of hypotensive epochs per hypotensive patient	2 (1–3) [0:6]		

*Note: Data are presented as n (%), mean ± standard deviation or median (interquartile range), [minimum : maximum]. CCI*, *Charlson Comorbidity Index; eGFR*, *estimated glomerular filtration rate; HbA1c*, *glycated haemoglobin; ICU*, *intensive care unit; INR, internal normalised ratio; pCO2*, *partial pressure of carbon dioxide; HDU*, *high dependency unit.*

^a^ *Defined as mean arterial pressure <65 mmHg or Systolic pressure <100* *mmHG*.

### Postoperative Complications

Overall, 45 patients (72.6%) experienced one or more postoperative complications during admission. Of these patients, 38 (84.4%) experienced between one and three complications, and seven (15.6%) had four or more complications. Regarding the severity of complications, 39 patients (86.7%) experienced either CVD grade I or II complications, 6 patients (13.3%) experienced CVD grade III complications and one patient (2.2%) experienced a CVD grade IV complication (see Table 2). No grade V complications were recorded. The types of complications are presented in Table 3.

**Table 2:** Postoperative complications and associated hospital costs.

Postoperative complications	Number	Hospital costs (USD) Median (IQR) Minimum: Maximum	*p*-value
*Patients without complications*	17 (27.4%)	30,946.9 (23,910.8–46,828.1) [16,672.5:106,850.5]	0.151^a^
*Patients with any complication*	45 (72.6%)	40,717.8 (27,358.0–59,834.3) [19,148.4:214,377.7]	
* Number of complications per patient*			
* Count of complications*	2 (1–3) [1:6]	–	*Not applicable*
** **1–3	38 (84.4%)	35,765.2 (26,306.5–54,004.5) [191,48.4:129,568.3]	0.015*^b^
** ** ≥4	7 (15.6%)	51,327.0 (49,201.5–140,980.6) [40,553.7:214,377.7]^†^	
* CVD grade of complication*			
** **I, II	39 (86.7%)	37,341.4 (26,462.1–51,327.0) [19,148.4:129,568.3]	0.007*^c^
** **III, IV	6 (13.3%)	109,051.3 (47,149.2–159,329.9) [40,992.4:214,377.7]^†^	

*Note: Data are presented as n (%) or median (interquartile range), [minimum : maximum]. CVD*, *Clavien–Dindo classification system.*

^*a*^ *Mann–Whitney U-statistic = 291.0;*

^*b*^ *Kruskal–Wallis one-way analysis of variance on ranks, H(2) = 8.404;*

^*c*^ *Kruskal–Wallis one-way analysis of variance on ranks, H(2) = 10.007*.

*p < 0.0167, Bonferroni’s adjustment.

†*p < 0.05 vs other two levels, Dunn’s all pairwise multiple comparison procedure.*

**Table 3:** Types of complications. (Some patients had a more than one complication).

Type of complication	Number of patients	Proportion
Cardiovascular		
Brady/tachycardia arrythmia requiring review	10	16.1%
Hypotension (volume depletion or vasoplegia) requiring treatment	8	12.9%
Congestive cardiac failure/fluid overload	1	1.6%
Myocardial infarction	0	0.0%
Pulmonary		
Atelectasis requiring oxygen/physiotherapy	8	12.9%
Pneumonia	6	9.7%
Pleural effusion	5	8.1%
Pulmonary embolus	1	1.6%
Respiratory failure mechanical ventilation	1	1.6%
Gastrointestinal		
Postoperative pancreatic fistula^a^ (total number of patients)	13	20.9%
Grade A	4	6.5%
Grade B	6	9.7%
Grade C	3	4.8%
Severe nausea and vomiting	8	12.9%
Ileus/delayed gastric emptying^b^ (total number of patients)	4	6.5%
Grade A	1	1.6%
Grade B	2	3.2%
Grade C	1	1.6%
Surgical site/wound complication	0	0.0%
Haematalogical		
Postoperative anaemia requiring treatment	2	3.2%
Thrombosis	0	0.0%
Renal		
Acute kidney injury	2	3.2%
Urinary tract infection	0	0.0%
Metabolic		
Endocrine derangement	16	25.8%
Electrolyte derangement	9	14.5%
Neurological		
Delirium/hallucinations	1	1.6%
Postoperative stroke/TIA	0	0.0%
Other		
Postoperative systemic inflammatory response syndrome	14	22.6%
Opioid side effect	1	1.6%
Dermatological	1	1.6%

^a^ *Postoperative pancreatic fistula: Grade A: no clinical symptoms but higher drain amylase levels; Grade B: clinical symptoms with radiographic imaging confirming peri-pancreatic fluid collections; Grade C: sepsis, organ dysfunction, and death*.

^b^ *Delayed gastric emptying: Grade A: nasogastric intubation lasting longer than postoperative day (POD) 3, reinsertion of nasogastric tube after the POD 3, or intolerance of solid diet by POD 7; Grade B: nasogastric intubation lasting for 8 to 14 PODs, the need to re-insert the nasogastric tube after POD 7 or intolerance to a solid diet by POD; Grade C: nasogastric intubation lasting for more than POD 14, reinsertion of nasogastric tube after POD 14, or intolerance of a solid diet by POD 21*.

There were no significant differences observed in the incidence of complications in patients who had a spleen preserving procedure vs. patients who underwent a splenectomy (*p *= 0.374). Similarly, there were no observed differences in the incidence of complication in patients who had open surgery vs. laparoscopic surgery (*p *= 0.313). Similarly, operating room expenditure, which including the costs of all surgical equipment, staplers, and disposables, was similar between patients with complications and those without complications ($14,433.6 [IQR:10,724.6–22,662.3] vs $11,627.8 [IQR 8,362.0–15,966.1]; *p* = 0.108). The median length of hospital stay was 9.1 days (IQR: 7.0–12.4). Patients with complications had a longer median length of stay compared to patients with no complications (7 days (IQR: 6.1–7.2) vs 10 (IQR: 7.1–12.8); *p* = 0.002).

### Unadjusted Hospital Cost

The median (IQR) cost (in USA dollars) of hospital stay was $30,946.9 (23,910.8–46,828.1) for patients who did not have complications vs. $40,717.8 (27,358.0–59,834.3) for patients who experienced complications (Mann–Whitney rank-sum test, *U* = 291.0, *p* = 0.151). This equated to a 31.6% increase in median cost between the two groups, however this was not statistically significant. Costs for patients with four or more complications were 43.5% higher costs than for those with three or fewer complications (Kruskal–Wallis *H*(2) = 8.404, *p* = 0.015). Further, the development of four or more complications was associated with a 65.8% increase in the cost compared to those patients who did not experience any complication.

**Figure 2:**
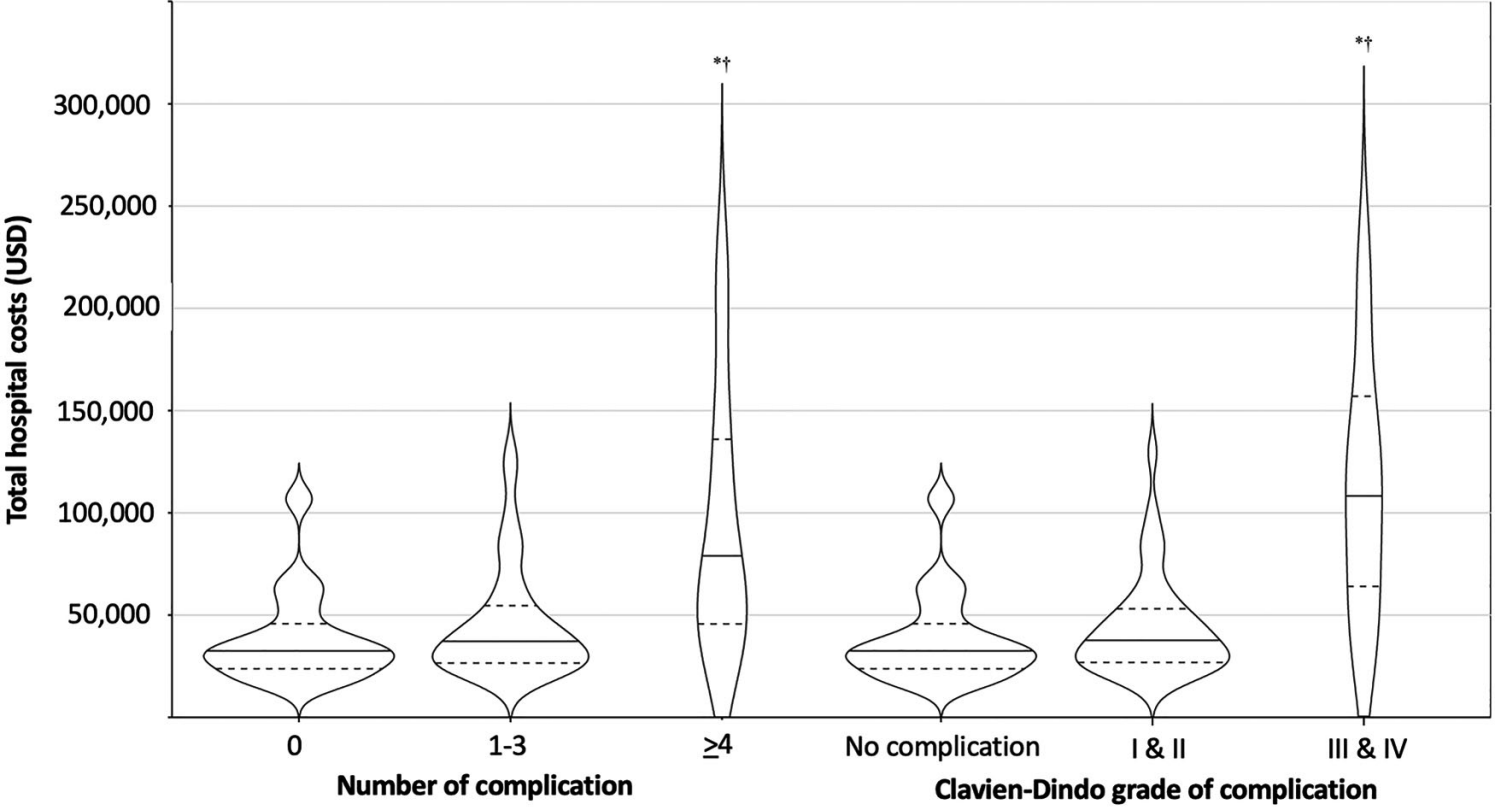
Unadjusted hospital cost according to the number and severity of complications. Horizontal lines and dashes indicate the median and dashes represent the 25%, and 75% cost of corresponding number and severity of complications. The width of each violine plot response to the approximated frequency in each region. *: Bonferroni’s adjusted *p* < 0.05 vs. no complications, ^†^: Bonferroni’s adjusted *p* < 0.05 vs. 1–3 complications or CVD I/II based on post-hoc Dunn’s test after the Kruskal–Wallis one-way analysis of variance on ranks.

**Figure 3:**
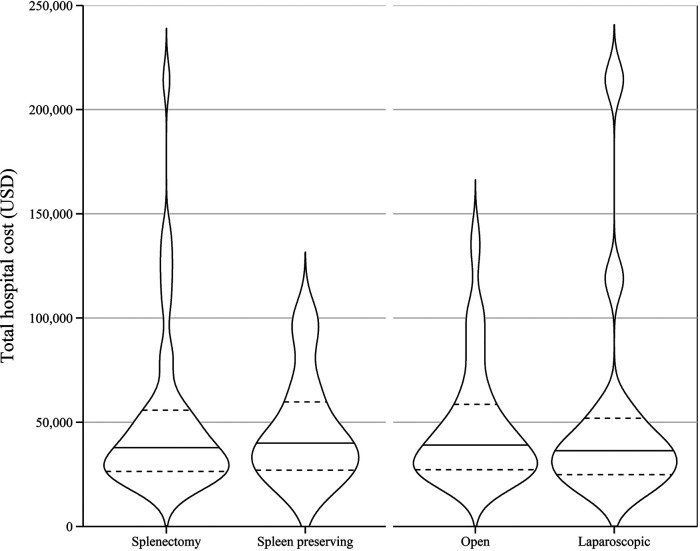
Unadjusted hospital cost of distal pancreatectomy with or without splenectomy and between open and laparoscopic surgeries.

**Figure 4:**
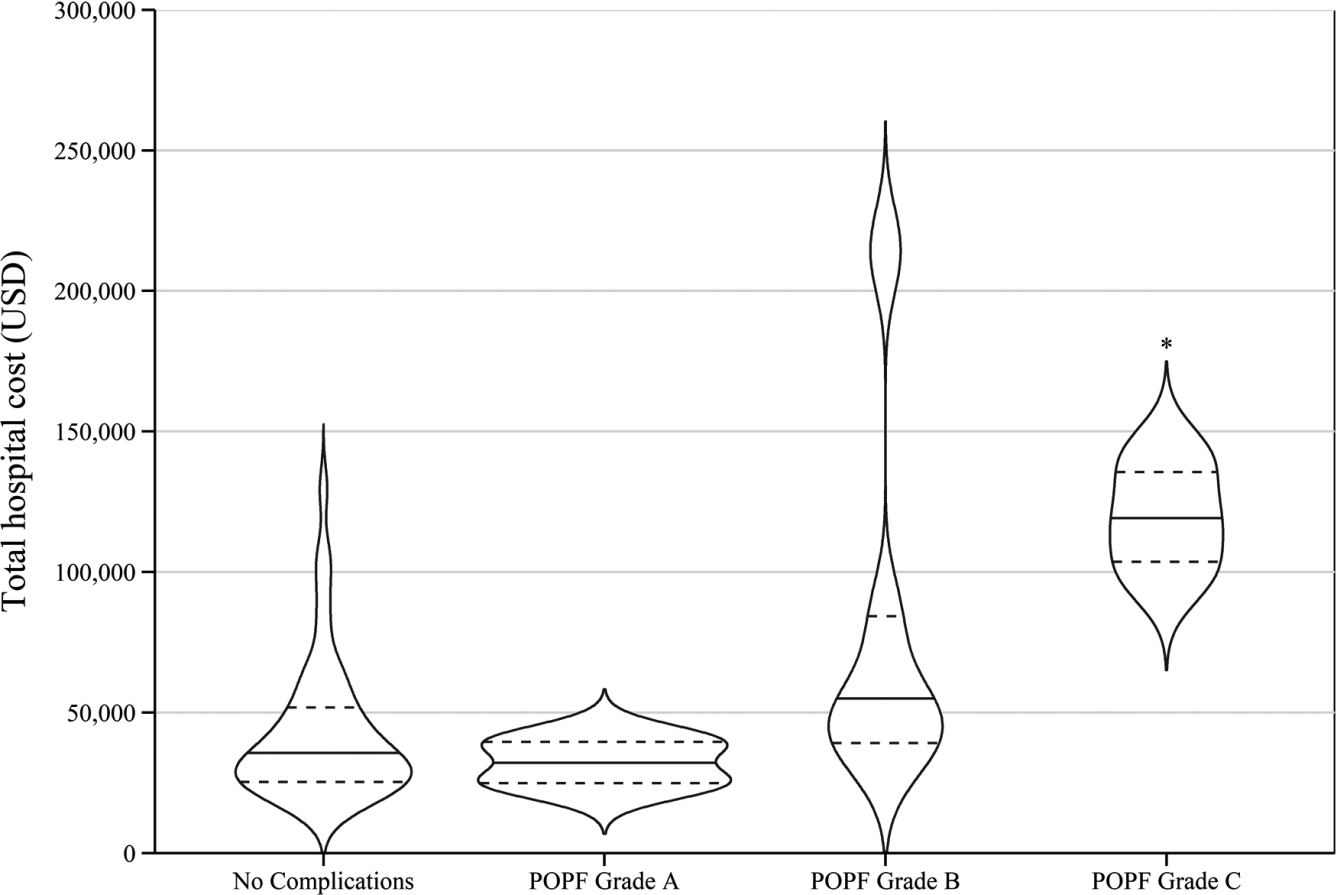
Unadjusted hospital cost according to the grade of postoperative pancreatic fistula. The pancreatic fistula is graded in agree with the International Study Group for Pancreatic Surgery definition of postoperative pancreatic fistula. POPF: postoperative pancreatic fistula. (*: *p* = 0.021 vs. no complications).

Regarding the severity of complications, there was no significant difference between the median costs for patients who had no complications and those who developed CVD grade I or II complications. However, the median cost for patients with CVD grade III/IV complications was significantly greater compared to those with either no complications or CVD grade I or II complications (Kruskal–Wallis *H*(2) = 10.007, *p* = 0.007). The unadjusted median costs according to number and severity of complications are presented in Table 2 and Figure 2. There were no significant differences observed in the unadjusted median costs between laparoscopic and open procedures (*p* = 0.635) or between spleen preserving and splenectomy procedures (*p* = 0.894) (Figure 3). The unadjusted median costs according to the grade of postoperative pancreatic fistula are presented in Figure 4.

### Adjusted Hospital Cost

Correlation analysis was performed for the collected variables to determine factors that were correlated with postoperative complications and hospital costs (Supplementary Table S1). Among the collected variables, coefficients that were either less than −0.2 or greater than 0.2 and that were also statistically significant were considered adjusting covariates for the regression model. A variable with a significant correlation coefficient was considered a candidate for the adjustment factor.

To reduce possible multicollinearity, some of the selected variables were excluded based on both clinical aspects and the correlation analysis results. The final selected adjusting factors were CCI, preoperative and postoperative haemoglobin concentration, white blood cell count, plasma bicarbonate, eGFR, postoperative lactate, duration of ICU admission, length of hospital stay, readmission and days alive and out of hospital.

Linear regression models were estimated with the variables of complications and selected factors (Table 4). During regression analysis modelling, complications themselves did not have a statistically significant effect on the hospital cost (regression coefficient of any complication = 6,379.9, *p* = 0.334, 95% CI, −6790.2–19,550.0), nor did the number of complications (coefficients of 1–3 complications = 5,878.6, *p *= 0.361, 95% CI, −6,966.9–18,724.0) or having four or more complications (≥4 complications = 20,154.3, *p* = 0.126, 95% CI, −5,881.1–46,189.7). However, CVD III/IV complications were found to cause a statistically significant increase in hospital costs, equating to $7,067.3 (*p* = 0.014, 95% CI, 12,047.7–102,087.0). Increased hospital costs were also strongly associated with readmission, irrespective of the presence, number or severity of patient complications.

**Table 4:** Adjusted hospital cost regression models.

Variables	Presence of complications		Number of complications		Severity of complications	
	Coefficient (95% CI)	*p*-value	Coefficient (95% CI)	*p*-value	Coefficient (95% CI)	*p*-value
(Constant)	229,682.9 (115,774.7–343,591.0)	<0.001*	229,264.8 (118,119.2–340,410.3)	<0.001*	248,228.4 (141,152.1–355,304.6)	<0.001*
CCI	1,578.2 (−767.3–3,923.7)	0.182	1,821.1 (−487.8–4,130.0)	0.119	2,246.2 (−86.3–4,578.6)	0.059
*Preoperative*						
Haemoglobin	−343.6 (−827.7–140.5)	0.160	−287.9 (−762.7–187.0)	0.228	−236.0 (−695.7–223.7)	0.306
White cell count	4,355.8 (2,040.7–6,670.9)	<0.001*	3,590.9 (1,209.3–5,972.5)	0.004*	2,074.7(−454.6–4,604.0)	0.105
Bicarbonate	−3,040.5 (−5,461.9–−619.2)	0.015*	−3,589.1 (−6,013.4–−1,164.8)	0.005*	−4,123.9 (−6,474.0–−1,773.8)	0.001*
eGFR	−415.0 (−1,029.3–199.3)	0.180	−339.3 (−958.8–280.2)	0.275	−276.3 (−864.9–312.3)	0.348
Duration of ICU admission	67.1 (−64.5–198.8)	0.310	211.9 (−95.4–519.1)	0.171	105.9 (−230.7–442.4)	0.529
*Postoperative*						
Haemoglobin	147.0 (−327.5–621.5)	0.536	102.3 (−373.6–578.1)	0.667	132.1 (−314.2–578.3)	0.553
White cell count	−1,469.7 (−2,726.7–−212.7)	0.023*	−1,174.7 (−2,459.3–109.8)	0.072	−404.4 (−1,815.1–1,006.4)	0.566
Bicarbonate	−586.3 (−3,528.4–2,355.8)	0.690	109.2 (−2,963.0–3,181.4)	0.943	−178.2 (−3,030.5–2,674.1)	0.900
eGFR	146.3 (−318.8–611.4)	0.529	107.5 (−353.0–568.1)	0.640	116.0 (−321.2–553.3)	0.595
Lactate	629.8 (−5,597.2–6,856.8)	0.839	104.3 (−6,285.7–6,494.3)	0.974	−1,901.3 (−7,974.2–4,171.5)	0.530
Days alive and out of hospital	−959.9 (−1,465.9–−454.0)	<0.001*	−1,006.7 (−1,501.8–−511.5)	<0.001*	−1,118.7 (−1,622.5–−614.9)	<0.001*
Readmission	21,426.9 (10,034.4–32,819.4)	<0.001*	18,671.3 (7,260.8–30,081.8)	0.002*	17,934.0 (6,705.8–29,162.3)	0.002*
Length of stay	702.3 (−248.0–1,652.6)	0.144	326.1 (−701.0–1,353.2)	0.525	−499.8 (−1,730.4–730.7)	0.417
*Complications*						
Any complication	6,379.9 (−6,790.2–19,550.0)	0.334				
1–3 complications			5,878.6 (−6,966.9–18,724.0)	0.361		
≥4 complications			20,154.3 (−5,881.1–46,189.7)	0.126		
CVD I					−4,574.8 (−24,060.4–14,910.8)	0.638
CVD II					11,663.2 (−1,292.1–24,618.4)	0.076
CVD III/IV					57,067.3 (12,047.7–102,087.0)	0.014*
*Model diagnostics*	*R*^2^ = 0.789, Durbin–Watson statistic = 1.815, *F*(3.97 × 10^9^,15) = 10.978, *p *< 0.001		*R*^2^ =0.796, Durbin–Watson statistic = 1 .883, *F*(3.52 × 10^9^, 16) = 10.257, *p* < 0.001		*R*^2^ = 0.826, Durbin–Watson statistic = 1.931, *F*(3.43 × 10^9^,17) = 11.163, *p *< 0.001	

*Note: Linear regression models were estimated using the hospital cost as the dependent variable. Significance testing of each model was evaluated with the Bonferroni’s corrected p-value <0.01667.*

*CCI*, *Charlson comorbidity index; CI*, *confidence interval; CVD*, *Clavien–Dindo grade of complication; eGFR*, *estimated glomerular filtration rate; ICU*, *intensive care unit*.

*Coefficient p-value <0.05.

## Discussion

### Key Results

We retrospectively analysed the complications and financial burden for patients undergoing a DP at a high-volume teaching hospital. In terms of complications, approximately three out of four patients experienced one or more postoperative complications during their admission. Compared to patients with no complications, the development of a minor complication (i.e., CVD grade I/II) increased the median hospital costs by 20.6%. However, patients who developed a major complication (i.e., CVD grade III/IV) had a 252% increase in median hospital costs with statistical significance. Our findings imply that the development of a major postoperative complication is the largest driver of hospital costs in patients undergoing distal pancreatectomy.

### Relationship to the Literature

Due to the considerable variability in the literature regarding the definition and recording of postoperative complications, it is difficult to compare our findings with other studies. Within the limited body of literature, some studies have investigated the incidence of only a single complication, such as postoperative diabetes (6) or pancreatic fistula (7, 19, 20) while other studies have focused on a select collection of major complications and omitted the record of minor complications in their final data (1, 21). As a reflection of the comprehensiveness of our data, since we elected to include all minor and major complications postoperatively, our complication rate is higher than those of the aforementioned studies. We elected to stratify all post-surgical complications using the CVD classification (18). A study by Lee et al. using the American College of Surgeons National Surgical Quality Improvement Program (ACS-NSQIP) database estimated complication rates of 27.0%. However, this figure cannot be directly compared with our complication rate as the study only identified and recorded 22 standardised postoperative complications (21). Studies might underestimate the rate of complications when omitting minor complications from their final statistical results. Reasons for omission may be due to the complications being regarded as clinically insignificant in predicting long-term morbidity or not directly resulting from the specific surgical procedure. de Rooij et al. found a post-laparoscopic DP complication rate of 16% and an open DP complication rate of 29%. Once again, only major complications (CVD grade ≥III) were included in their analysis, hence the lower rate of reported complications (22). However, these figures are in fact consistent with the percentage of patients who experienced major complications (CVD grade III/IV) in our cohort (1 in 10 patients) and, thus, support the validity and precision of our results.

Overall, the number of complications experienced by a patient did not significantly add to hospital costs compared to patients with no postoperative complications. However, we found that hospital costs were significantly increased (by one and a half times) when patients suffered four or more postoperative complications compared to patients with fewer than four complications. Conversely, experiencing a major complication requiring surgical, endoscopic, or radiological intervention (CVD grade III) had an additive effect on hospital financial expenses. Costs increase for these patients was almost three times higher than for patients with CVD grade II complications and five times higher for patients with no complications. From our data, we suggest that hospital costs are primarily driven by major complications rather than the number of complications following DP. This correlation between major complications (CVD grade ≥III) and additional financial burden is consistent with findings from several other studies analysing costs associated with post-abdominal surgery complications (12, 14, 23). However, these studies also found a significant association between the cumulative number of complications and pro-rata cost increases, in contrast to the findings of our study which did not demonstrate a statistically significant link.

Although there is little research comparing the morbidity and mortality rates for different surgical procedures between different specialties, Cardini et al. found DP to have the lowest morbidity risk and length of hospital stay compared to other pancreatic resection techniques (24). These findings suggest that, generally, the complications of patients following DP may be less severe than those of other surgical procedures, irrespective of the number of complications. This is further supported by our data suggesting that minor postoperative complications (CVD grade I/II) did not affect healthcare costs as much as major complications.

The additional costs incurred from major complications (CVD grade III/IV) are largely attributed to the need for readmission and procedural requirements to treat the complications, including staffing, equipment and ICU costs. Readmission was an independent factor in increasing adjusted additional costs. Readmission following pancreatectomy has also been associated with earlier patient mortality (9). Thus, not only does the prevention of major complications benefit a hospital financially, the health implications of early recognition of patient deterioration affect patients’ long-term prognosis and quality of life. Kennedy et al. investigated the implementation of critical pathways (or fast-track protocols) in streamlining and systematising patient care for those undergoing a DP. By setting a structured multidisciplinary care pathway in a major teaching hospital, rates of readmission were reduced by over 50%. Hospital costs were also significantly reduced (25). Our research encourages the implementation of more cost-efficient hospital protocols, such as the aforementioned intervention, to reduce the severity of patient complications, length of hospital stay and rates of readmission. Finally, less than a quarter of patients in our study underwent laparoscopy surgery. Laparoscopic DP (compared to open), maybe associated with less postoperative pancreatic fistula (26), a tendency for lower blood loss, reductions in operative trauma and duration of postoperative recovery without compromising the oncologic resection (27), and shorter hospital stay (27–29), hence lower costs. The observed outcomes of laparoscopic DP are promising, and further studies are required to comprehensively assess its cost effectiveness.

### Strengths and Limitations

There are several strengths to our study. We have provided a comprehensive analysis of the relationship between postoperative complications, including the quantity and severity of such complications and hospital costs following DP resection surgery. We used the CVD system to classify the severity of postoperative complications. First described in 2004 (18), this classification system is standardised, validated and internationally recognised (30). Consequently, the complications described in our research can be easily compared to those in other current and future studies. Further, our study focused on all complications, regardless of type and severity, with data analysed from a comprehensive hospital cost database. This is also one of the first studies to use regression analysis to account for multiple variables and factors that may be associated with the hospital cost of complications.

Our study has several limitations. The patient data collected was only from a single university hospital. This may limit the study’s external validity and extrapolative potential. Although the study was conducted in a high-volume centre with surgical and anaesthetic protocols similar to those in other tertiary centres, there is a relatively low number of patients. In addition, less than a quarter of patients underwent laparoscopic surgery. We preference an open approach when there are concerns from preoperative imaging that there may be invasion of surrounding organs or critical vasculature, or distant metastasis, and for radical cancer operations. Whilst obesity, the elderly and the very frail patient are not contra-indications to a laparoscopic approach, in our experience, in such settings laparoscopic surgery is more challenging, and an open approach is preferred. Finally, we consider an open approach if preservation of the short gastric vessels is imperative.

The study was retrospectively, which may confer a degree of selection and information bias. However, biases were limited through the employment of two authors to cross-check health records and incoming data. Finally, our study only accounted for short-term costs related to the hospital. We did not consider costs incurred in the long term or in the community, which could be an area for further expansion in future research.

### Conclusion

Understanding the financial implications of postoperative complications after DP is important for improving patient care while ensuring that health care is financially viable. The increase in the number and severity of postoperative complications in this study’s cohort significantly increased patients’ hospital costs. The development of a major postoperative complication was the largest driver of hospital costs. Building on this research will enable hospitals to employ targeted quality-improvement activities to enhance patient care and reduce expenditure.

## Data Availability

The raw data supporting the conclusions of this article will be made available by the authors, without undue reservation.
