# Supplementary material for: The Association of Postoperative Complications and Hospital Costs Following Distal Pancreatectomy
Source: Front Surg. 2022 May 30;9:890518. doi: 10.3389/fsurg.2022.890518 (PMC9195500; doi:10.3389/fsurg.2022.890518)
Supplement: Supplementary file 1 [file Data_Sheet_1_v1.pdf]

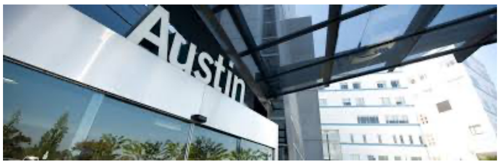

A/Prof Laurence Weinberg
Consultant Anaesthetist, Cardiac Anaesthesia and Hepatobiliary Surgery

Director
DEPARTMENT OF ANAESTHESIA
AUSTIN HEALTH

Tel: (03) 9496 3800 Fax: (03) 9459 6421
Email: Laurence.Weinberg@austin.org.au

Director of Anaesthesia
A/Prof Laurence Weinberg
BSc, MBBCh, MRCP, FANZCA, Dip.Crit.CareEcho, MD, PhD

Admin Managers
Mrs Val Hocking

Deputy Director (Admin)
Dr Dean Cowie
MBBS, FANZCA, Dip.Crit.CareEcho

Deputy Director (Clinical Operations):
Dr Louise Ellard
MBBS FANZCA
AdvPTEeXAM

The Surgical Centre
Head: Dr David Tremewen
MBBS, FANZCA, Dip.Crit.CareEcho,
Deputy Director: Dr Brett Pearce MBBS, FANZCA

Consultant staff Anaesthetists
Dr Daniel Banyaz
MBBS, FANZCA
Dr Tim Coulson
MBBS, FANZCA, PhD
Dr Stephanie Chen
MBBS, FRCA, FANZCA
Dr Justin Nazareth
MBBS, FANZCA
Dr Ranj Guha
MBBS, FANZCA
Dr Matt Lee
MBBS, FANZCA, AdvPTeXAM
Dr Charlotte Heldreich
MBBS, FANZCA
Dr David Lam
MBBS, FANZCA
Dr Stiofan O'Conghaile
MBBS, FANZCA, FFPMANZCA
Dr Ilonka Meyer
MBChB, PDD, FANZCA, FFPMANZCA
Dr Dishan Chandrasekara
MBBS, FANZCA
Dr Chong Tan
MBBS, FANZCA
Dr Raymond Hu
MBBS, FANZCA
Dr Audrey Bren
MBBS, FANZCA
Dr Lachlan Miles
MBBS, FANZCA, PGCertCU, AdvPTeXAM
Dr Jonathan Graham
MBBS, FANZCA
Dr Ian Harley
MBBS, FANZCA, FRCA
Dr Matt Lee
MBBS, BMedSci, FANZCA
Dr Antony Leaver
MBBS, FANZCA
Dr Frank Liskaser
MBBS, FANZCA
Dr Shiva Malekzadeh
MBBS, FANZCA
Dr Peter McCall
MBBS, FANZCA
Prof Philip Peyton
MBBS, FANZCA, DA(UK), DipObs(RACOG), MD, PhD
Dr Param Pillai
MBBS, MD Anaes, FRCA
Prof David Story
MBBS, FANZCA, MD
Dr Fiona Desmond
BMedSci, MBBCh, BAO(Hon), FCARSI, FJFICMI, DipPianMed, FANZCA

Professor Andrew Gumbs MD, FACS, FECS (Hon.)
Associate Editor
Frontiers in Surgery

Re: Manuscript title: The association of postoperative complications and hospital costs following distal pancreatectomy
Manuscript ID: 890518

On behalf of my co-authors, we would like to thank you and the Reviewers for considering our manuscript for publication in *Frontiers.* We appreciate the positive and constructive comments made.

Please find below a detailed response to the comments from the Expert Reviewers.

REVIEWER No 1 Questions

Reviewer 1 | 08 Mar 2022 | 12:20. This is a retrospective single center study aiming to evaluate the complications cost of DP. The issue is interesting as costs are directly related to complications and knowing its impact can improve cost efficiency. The methodology seems well shaped according to current international criteria. There are only few suggestions to the authors in order to improve the strength of their study.
Authors response: Thank you for your positive comments and for reviewing our manuscript. Your excellent and insightful comments and suggestions below are appreciated. Please find a detailed response to each of the questions you have raised.

Question 1: There are some few typesetting errors such as: "Lapracopic and open procedures were included"
Authors response: Thank you for pointing out these typographical errors and we are sorry that these were not previously corrected. The entire manuscript has been carefully checked and the typographical errors have been now corrected.

Question 2: Conversion-specific cost of the laparoscopy group might give more insight.
Authors response: Thank you for this excellent comment. In our manuscript, most patients (n=48, 77.4%) underwent open surgery. Less than a quarter of patients (n=14, 22.6% of all patients) underwent laparoscopy surgery. There were no conversions from laparoscopic to open, which we think is firstly the result of the relatively low proportion of patients who underwent laparoscopic surgery, but also careful patient selection. This detail has been added into the manuscript. We have also outlined in more detail in our response to Reviewer 2 below (Question 1) the reasons that our centre undertakes a relatively low number of laparoscopic procedures for distal pancreatectomy compared to open procedures.

Question 3: Cost of the specific complication of pancreatic fistula only may be interesting to understand its impact on DP.
Authors response: This is an excellent comment, which we have now investigated in detail and reported in the resubmission manuscript. We do however express caution when interpreting our results, given the low numbers of patients in each group; we acknowledge the fact that the more inferences that we make, the higher the chances of Type 1 error.

Table 1 and **Figure 1** below, summarises the association of costs severity of POPF. Interestingly, and not unexpectedly, there were no clinically or statistically significant increases in cost with a Grade 1 POPF compared to patients with no complications (p=0.99), given that the definition of a Grade is “no clinical symptoms but higher drain amylase levels”. However, patients with Grade B POPF and Grace C had a clinically important increase in costs when compared to patients without any complication.

We have now included this addition Figure in the manuscript. Thank you once again for this excellent suggestion.

Table 1: Unadjusted hospital cost in USD according to the postoperative pancreatic fistula grade (POPF) . (*P<0.021 Grade III vs. no complications).

	**No complications (N=17)**	**Grade I POPF (N=4)**	**Grade II POPF (N=6)**	**Grade III POPF (N=3)**
**Median**	$30,946.93	$31,886.85	$50,158.51	$119,053.43*
**25th**	$23,910.80	$24,583.39	$39,750.60	$99,049.22
**75th**	$46,828.16	$40,227.83	$116,144.28	NA
**Min**	$16,672.53	$24,164.66	$37,341.40	$99,049.22
**Max**	$106,850.53	$40,992.40	$214,377.69	$140,980.60

Figure: Unadjusted hospital cost according to the postoperative pancreatic fistula. The pancreatic fistula is graded in agree with the International Study Group for Pancreatic Surgery definition of postoperative pancreatic fistula. POPF: postoperative pancreatic fistula. *: P<0.021 vs. No complications.

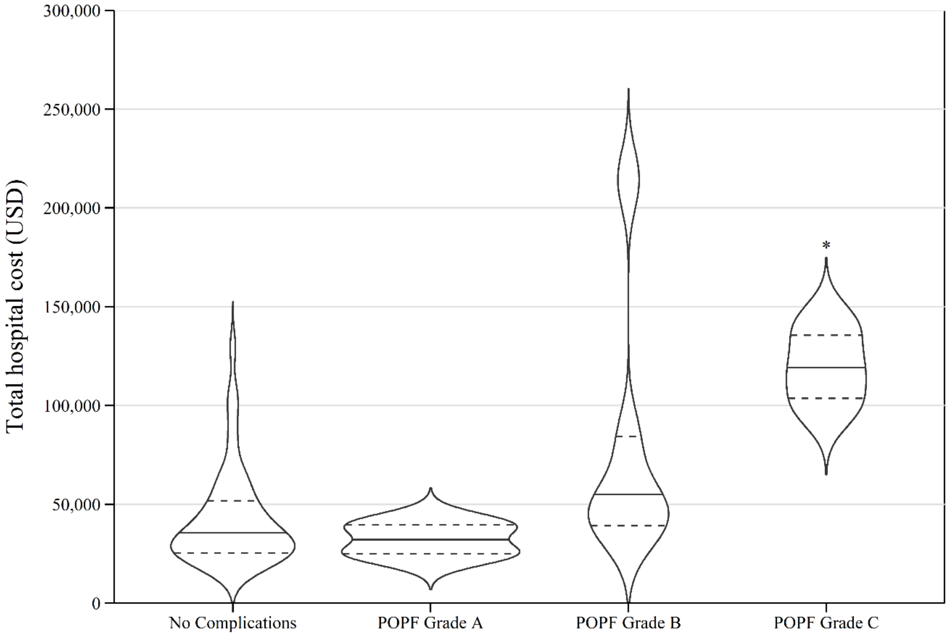

Question 4: Laparoscopic vs open approach cost differences, as well, may be interesting. Some further sub analysis of spleen preserving as well may be useful for the final analysis.

Authors response: Thank you again for this excellent comment. Once again, we now present the costs of laparoscopic vs. open and well as spleen preserving vs. splenectomy. The Figure below, graphically addresses this important question and displays the unadjusted hospital cost according to the surgical techniques. The left column of graph presents hospital cost of pancreatectomy with or without splenectomy, and the right column shows the hospital cost between open and laparoscopic surgery. We were no statistically significant differences in costs observed between splenectomy and spleen preserving surgeries between and open vs. laparoscopic. (Mann-Whitney U test, U = 421, p-value = 0.894 between splenectomy and spleen preserving. U = 365, p-value = 0.635 between open vs. laparoscopic).

This Figure has been included in the revised manuscript. Thank you for this excellent suggestion.

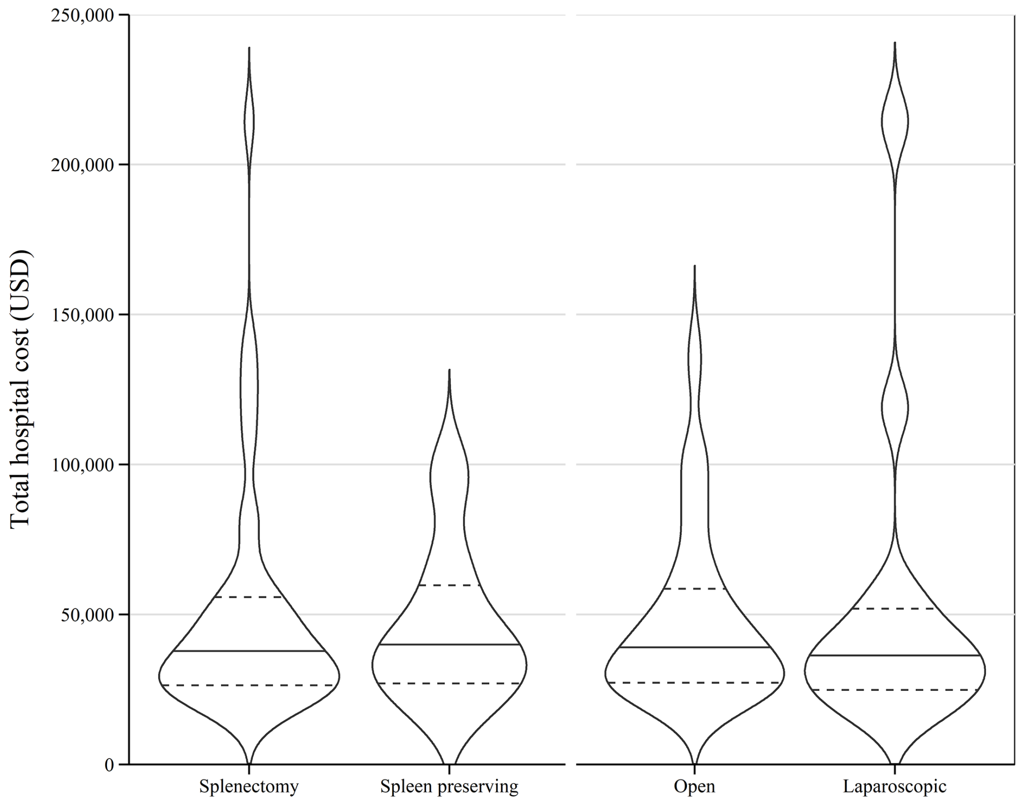

Question 5: I cannot easily understand which are the operative costs of the operative time, and operative devices (Stapler, energy devices). This data is important to check how they might affect results.

Authors response: Thank you for you for this insightful comment which we have now included in the revised manuscript. We now state *"Similarly, operating room expenditure, which including costs of surgical equipment, staplers, and disposables was similar between patients with complications and those without complications ($14,433.6 [IQR:10,724.6-22,662.3] vs $11,627.8 [IQR 8,362.0-15,966.1]; p=0.108).*

REVIEWER NO 2 QUESTIONS

Reviewer 2 | 15 Mar 2022 | 16:19. Interesting cost evaluation work for a specific surgical procedure (distal pancreatectomy). The authors manage to verify, with a good statistical analysis, the relationship between complications and increases in hospital costs. The patient population is homogeneous in terms of variables evaluated. Logically, costs increased considerably depending on the degree of complication. When they are type 3 or 4, invasive procedures are required, which therefore increase costs.
Authors response: Thank you for your positive comments and we appreciate the time you have taken to review our manuscript.

Question 1. The low number of laparoscopic procedures for the distal pancreas, requires a comment considering that today is the election procedure.
Authors response: Thank you for this excellent comment.

In the revised manuscript we now state *"In addition, less than a quarter of patients underwent laparoscopic surgery. We preference an open approach when there are concerns from preoperative imaging that there may be invasion of surrounding organs or critical vasculature, or distant metastasis, and for radical cancer operations. Whilst obesity, the elderly and the very frail patient are not contra-indications to a laparoscopic approach, in our experience, in such settings laparoscopic is more challenging and an open approach is preferred. Finally, we consider an open approach if preservation of the short gastric vessels is imperative."*

We agree that laparoscopic distal pancreatectomy should theoretically provide the same postoperative recovery advantages reputed to minimal access surgery, however our unit has had concerns as to the safety of laparoscopic distal pancreatectomy in terms of life-threatening intraoperative events, adequate oncological outcomes as compared to the traditional “open” distal pancreatectomy, as well as the safety challenges with a “minimal access approach” is the very obese, the elderly or the frail.

In our centre, we do consider that the indications for laparoscopic distal pancreatectomy are indeed very similar for open. We tend to consider laparoscopic distal pancreatectomy for benign, borderline, or malignant tumours of the pancreatic body and tail, however we advocate for open procedures when there are concerns from imaging that there may be invasion of surrounding organs or critical vasculature, distant metastasis in cancer, or acute pancreatitis and for radical cancer operations. Whilst obesity, the elderly and the very frail patient is not a formal contra-indication, in our experience laparoscopic distal pancreatectomy is more challenging, and an open approach is often preferred. We also agree that spleen preserving techniques may be easier to perform laparoscopically, however the preservation of the short gastric vessels, which is required in the Warshaw technique, might be more complex to perform laparoscopically. We certainly acknowledge that more centers are adopting the laparoscopic technique for technique distal pancreatectomy (as is our centre), however at present we think there is a lack of compelling level 1 evidence supporting laparoscopic distal pancreatectomies for all cases. Therefore, we embrace this technique selectively.

Current supporting evidence for this laparoscopic distal pancreatectomy exists can be found in retrospective case series and a few case-control studies.[1-5] Notable is the recent work of Kooby et al.,[4] a large multi-institutional case-control study in which 142 laparoscopic distal pancreatectomies were compared with 200 open distal pancreatectomies. Patients were matched based on age, pathologic findings, ASA criteria, and pancreatic specimen length. Similar to our findings, there was no increase in major morbidity or pancreatic leak rate with the laparoscopic approach. Their reported mortality was also 0%. We also acknowledge that many of the retrospective studies are limited by their multi-institutional, retrospective nature and the between centre variability.

In 2012, a systematic review and meta-analysis by Venkat at al.[6] reported that laparoscopic distal pancreatectomy (compared to open, all other factors being equal) was associated with less postoperative pancreatic fistula. Similarly, Khaled et al.[7] reported that the laparoscopic approach to distal pancreatectomy offers advantages over open surgery in terms of reductions in operative trauma and duration of postoperative recovery without compromising the oncologic resection. This was a single centre retrospective case-matched observational study.

More recently however, Røsok et al.[8] evaluated 582 studies, 52 (40 observational and 12 case-matched) were included in the assessment for outcome for laparoscopic distal pancreatectomy (n = 5023) vs. open (n = 16,306) whereas 16 observational comparative studies were identified for cancer outcome. No randomized trials were identified. The authors concluded that there was a tendency for lower blood loss and shorter hospital stay in the laparoscopic group, however they stated that available evidence for comparison of laparoscopic to open is “weak”, although the number of studies is high and the observed outcomes of laparoscopic surgery are “promising”, in the absence of randomized control trials, an international registry should be established. We agree with this statement.

In the 2016 Cochrane review by Riviera et al.[9] who examined laparoscopic versus open distal pancreatectomy for pancreatic cancer, it was reported that there is a dearth of randomised controlled trials that have compared laparoscopic distal pancreatectomy versus open distal pancreatectomy for patients with pancreatic cancers. This review did report that laparoscopic distal pancreatectomy has been associated with shorter hospital stay when compared with open distal pancreatectomy, however they also stated that currently, no information is available to determine a causal association in the differences between laparoscopic versus open distal pancreatectomy.

We also appreciate that the observed differences may be a result of confounding due to laparoscopic operation on less extensive cancer and open surgery on more extensive cancer. In addition, differences in length of hospital stay are relevant only if laparoscopic and open surgery procedures are equivalent from a positive oncological outcome perspective. This information is not available currently. Thus, randomised controlled trials are needed to compare laparoscopic distal pancreatectomy versus open distal pancreatectomy with at least two to three years of follow-up. Such studies should not only include postoperative complications (as we have reported), but patient-oriented outcomes, long-term mortality (at least two to three years), health-related quality of life, resection margins, and of course recurrence of cancer.

Question 2. The strength of the study is to be able to demonstrate with a good analysis the considerable increase in costs in relation to postoperative complications. One of the weaknesses is not commenting on actions to try

to reduce costs. For example: increase mini-invasive surgeries with fewer days of hospitalization, actions to reduce incidences of pancreatic fistulas, etc.
Authors response: Thank you for this excellent and insightful comment, which we have now addressed in the discussion section.

We state "*Finally, less than a quarter of patients in our study underwent laparoscopy surgery. Laparoscopic DP (compared to open), maybe associated with less postoperative pancreatic fistula,*[26] *and a tendency for lower blood loss, reductions in operative trauma and duration of postoperative recovery without compromising the oncologic resection,*[27] *and shorter hospital stay,*[27-29] *hence lower costs. The observed outcomes of laparoscopic surgery are promising, and further studies are required to comprehensively assess its cost effectiveness.*

Four additional refences have been added to support this statement.

Question 3. Tables 1 and 4 are very long and difficult to follow. Could be summarized
Authors response: Thank you these comments. Table 1 has been significantly shortened and we have deleted all variables that are not directly relevant to the manuscript.

We agree with the experts Reviewers regarding Table 4. This table has now been removed from the main manuscript and included as a supplementary file.

Once again, thank you for the time taken to review our revised manuscript.

L Weinberg

A/Prof Laurence Weinberg
BSc, MBBCh, MRCP, DPCritCareEcho, FANZCA, MD, PhD

Director, Department of Anesthesia, Austin Hospital, Australia
A/Professor, Department of Critical Care, The University of Melbourne, Australia

References

1. Melotti G, Butturini G, Piccoli M, Casetti L, Bassi C, Mullineris B, Lazzaretti MG, Pederzoli P. Laparoscopic distal pancreatectomy: results on a consecutive series of 58 patients. Ann Surg. 2007 Jul;246(1):77-82.

2. Nakamura Y, Uchida E, Aimoto T, Matsumoto S, Yoshida H, Tajiri T. Clinical outcome of laparoscopic distal pancreatectomy. J Hepatobiliary Pancreat Surg. 2009;16(1):35-41. doi: 10.1007/s00534-008-0007-0. Epub 2008 Dec 16. PMID: 19083146

3. Pryor A, Means JR, Pappas TN. Laparoscopic distal pancreatectomy with splenic preservation. Surg Endosc. 2007 Dec;21(12):2326-30. doi: 10.1007/s00464-007-9403-9. Epub 2007 Jun 26. PMID: 17593458.

4. Kooby DA, Gillespie T, Bentrem D, Nakeeb A, Schmidt MC, Merchant NB, Parikh AA, Martin RC 2nd, Scoggins CR, Ahmad S, Kim HJ, Park J, Johnston F, Strouch MJ, Menze A, Rymer J, McClaine R, Strasberg SM, Talamonti MS, Staley CA, McMasters KM, Lowy AM, Byrd-Sellers J, Wood WC, Hawkins WG. Left-sided pancreatectomy: a multicenter comparison of laparoscopic and open approaches. Ann Surg. 2008 Sep;248(3):438-46. doi: 10.1097/SLA.0b013e318185a990. PMID: 18791364

5. Eom BW, Jang JY, Lee SE, Han HS, Yoon YS, Kim SW. Clinical outcomes compared between laparoscopic and open distal pancreatectomy. Surg Endosc. 2008 May;22(5):1334-8. doi: 10.1007/s00464-007-9660-7. Epub 2007 Nov 20. PMID: 18027035.

6. Venkat R, Edil BH, Schulick RD, Lidor AO, Makary MA, Wolfgang CL. Laparoscopic distal pancreatectomy is associated with significantly less overall morbidity compared to the open technique: a systematic review and meta-analysis. Ann Surg. 2012 Jun;255(6):1048-59. doi:

7. Khaled YS, Malde DJ, Packer J, De Liguori Carino N, Deshpande R, O'Reilly DA, Sherlock DJ, Ammori BJ. A Case-matched Comparative Study of Laparoscopic Versus Open Distal Pancreatectomy. Surg Laparosc Endosc Percutan Tech. 2015 Aug;25(4):363-7. doi: 10.1097/SLE.0000000000000179. PMID: 26121539.

8. Røsok BI, de Rooij T, van Hilst J, Diener MK, Allen PJ, Vollmer CM, Kooby DA, Shrikhande SV; Organizing Committee for the State of the Art Conference on Minimally Invasive Pancreas Resection. Minimally invasive distal pancreatectomy. HPB (Oxford). 2017 Mar;19(3):205-214. doi: 10.1016/j.hpb.2017.01.009. Epub 2017 Feb 16. PMID: 28215903

9. Riviere D, Gurusamy KS, Kooby DA, Vollmer CM, Besselink MG, Davidson BR, van Laarhoven CJ. Laparoscopic versus open distal pancreatectomy for pancreatic cancer. Cochrane Database Syst Rev. 2016 Apr 4;4(4):CD011391.
